# Lichen striatus as an immune-related adverse event following ipilimumab/nivolumab and COVID-19 infection in an adult

**DOI:** 10.1016/j.jdcr.2024.05.038

**Published:** 2024-06-15

**Authors:** Courtney M. Kenyon, Brenna G. Kelly, Anneli R. Bowen, Matthew Gumbleton, Dekker C. Deacon

**Affiliations:** a University of Utah Spencer Fox Eccles School of Medicine, Salt Lake City, Utah; b Department of Dermatology, University of Utah, Salt Lake City, Utah; c Division of Oncology, Department of Internal Medicine, University of Utah School of Medicine, Salt Lake City, Utah; d Huntsman Cancer Institute, Salt Lake City, Utah

**Keywords:** COVID-19, immune checkpoint inhibitor, immune-related adverse event, immunotherapy, lichen striatus, rash, viral infection

## Introduction

The mechanisms governing the development of lichen striatus (LS) have yet to be fully elucidated; however, there have been links to immune stimulation, particularly T cell-mediated processes. Here, we report a presentation of adult-onset LS following the initiation of immune checkpoint inhibitor (ICI) therapy and concomitant COVID-19 infection.

## Case report

**Fig 1:**
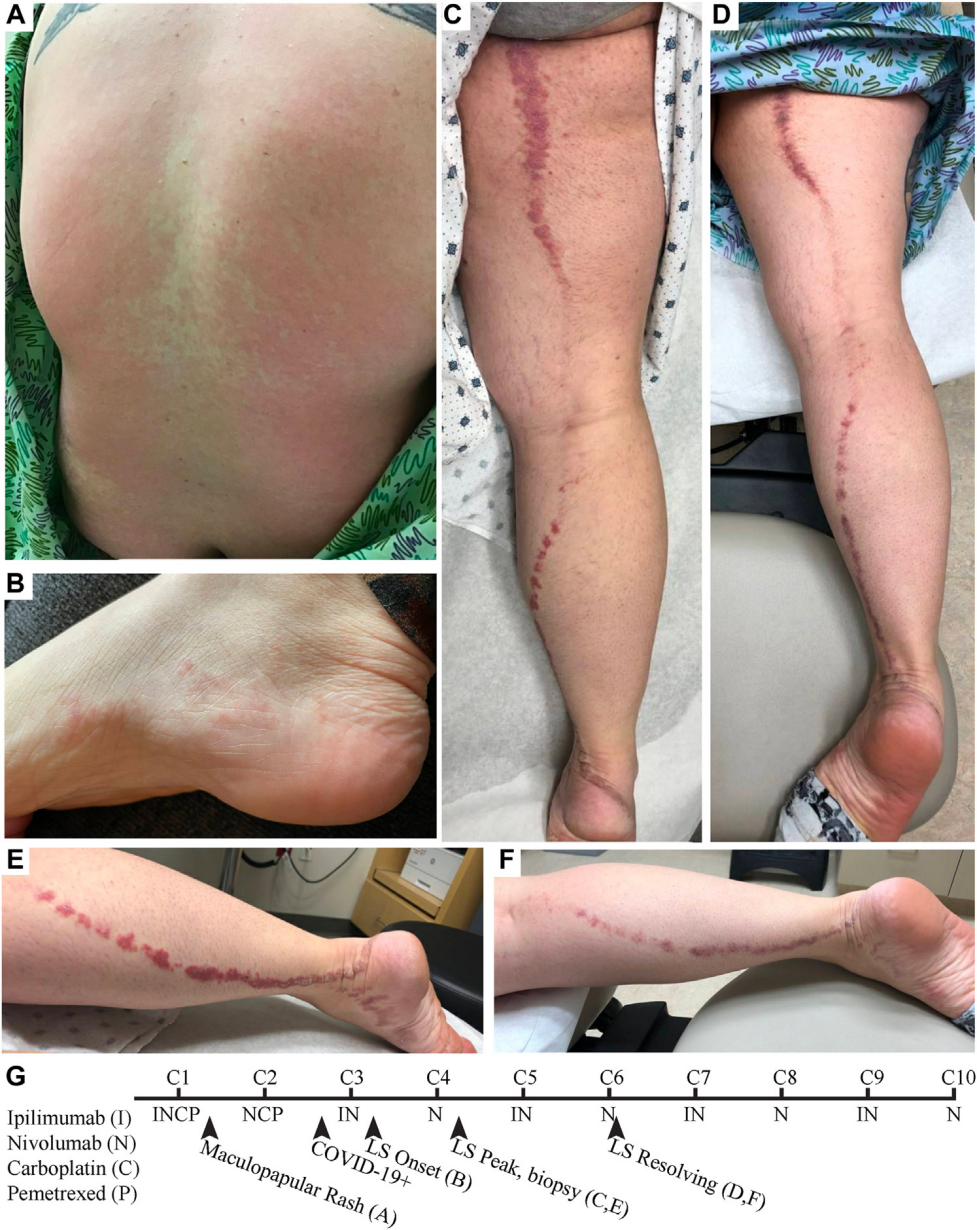
Maculopapular eruption that appeared 10 days into cycle 1 of CheckMate 9LA (**A**). Initial onset of *pink* papules at *right* instep following third cycle of CheckMate 9LA (**B**). Blaschkoid distribution of *purple* scaly plaques shortly after cycle 4 (**C** and **E**) and again after 6 weeks of intermittent topical triamcinolone use (**D** and **F**). Timeline of chemotherapy regimen and rash progression (**G**).

A 29-year-old Caucasian female with recently diagnosed stage IV invasive mucinous adenocarcinoma of the lung presented to dermatology for a rash on her right leg that appeared shortly after her third ICI infusion on cycle 3, day 2 (C3D2) of the CheckMate 9LA regimen (Fig 1, *G*).

Before presenting to dermatology, the patient had experienced a prior – and likely unrelated – pruritic rash located on the scalp, trunk, and extremities that appeared on C1D10. This eruption was diagnosed as a morbilliform rash (Fig 1, *A*), and the patient was treated with topical triamcinolone 0.1% cream and oral antihistamines with complete resolution. The patient tolerated her second infusion without any dermatologic complications. Notably, she began to experience COVID-19 symptoms about 9 days prior to her third infusion and tested positive shortly thereafter. Her symptoms were mild, and she recovered at home without complications. The patient then received her third infusion, after which she developed a second distinct rash.

This mildly-itchy rash first appeared on the instep of her right foot 2 days after her third infusion, and then spread up the back of her right leg (Fig 1, *B* and *C*). She denied fevers or systemic symptoms. She started triamcinolone 0.1% cream twice daily for 2 weeks without improvement.

**Fig 2:**
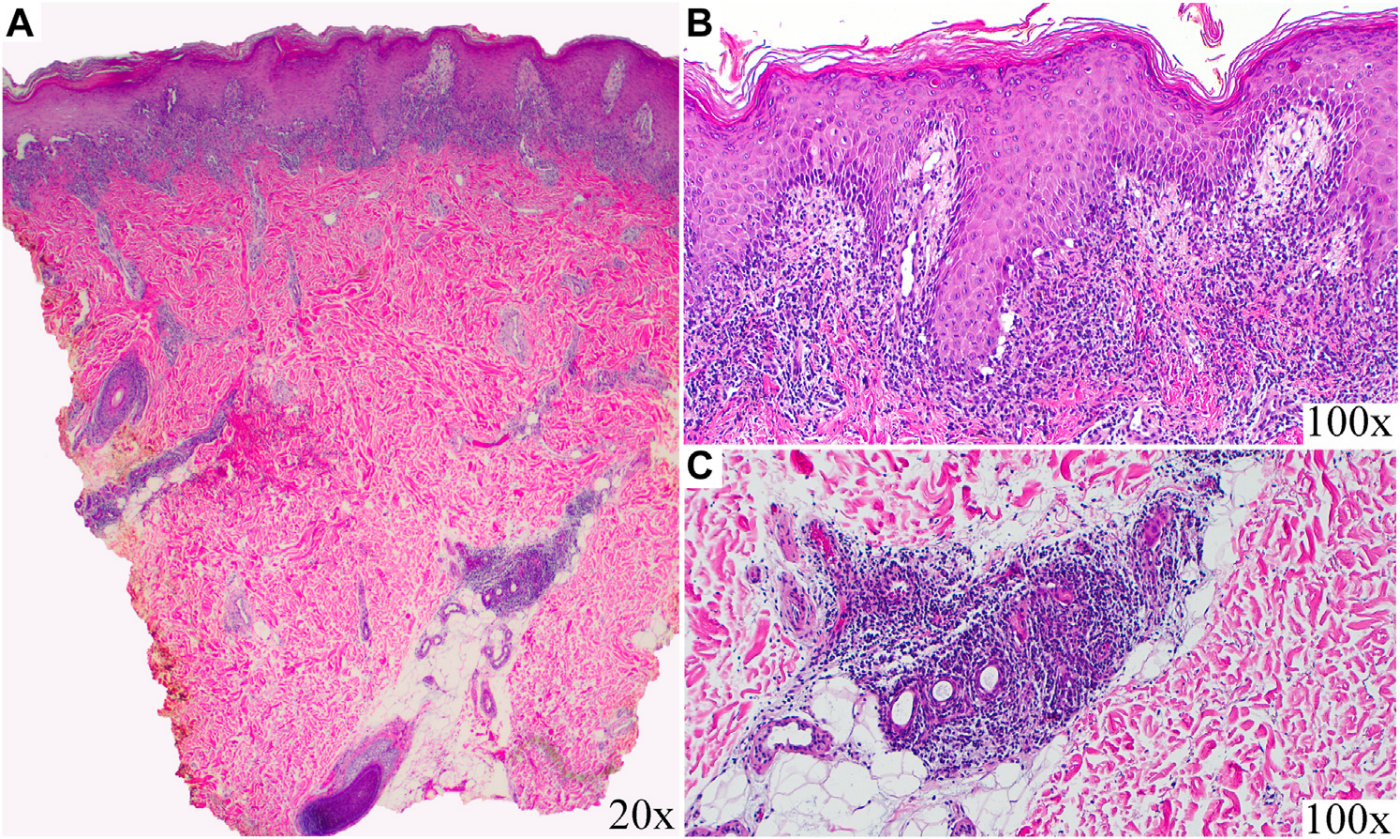
Low power view of a punch biopsy (**A**) of the right upper thigh revealed a lichenoid interface reaction pattern as well as deep perieccrine inflammation (20×) with routine hematoxylin and eosin staining. High power view of lichenoid infiltrate (**B**) and lymphocyte-predominate perieccrine infiltrate (**C**) (100×).

On initial dermatologic consultation, there was a dull, purple plaque that extended from the right foot to the right buttock in a blaschkoid distribution (Fig 1, *C*). Some overlying scale was present, primarily over the right heel (Fig 1, *E*). After examination, a 5 mm punch biopsy from the right upper thigh was obtained. Histopathologic analysis revealed a lichenoid tissue response with deep perieccrine inflammation consistent with a diagnosis of LS (Fig 2). She continued topical triamcinolone twice daily and, after almost 6 weeks of intermittent use, demonstrated clinical improvement. Triamcinolone was discontinued, and she completed CheckMate 9LA cycles 6 – 10 without rash reoccurrence.

## Discussion

LS is an acquired, self-limiting inflammatory skin eruption. The exact incidence is unknown, however, it is rare in adults and most commonly affects children between the ages of 5 to 15 years.^1^ LS typically presents as flat-topped papules and plaques along the lines of Blaschko, usually on an extremity. In prior cases, adult LS has been temporally associated with trauma, vaccines, medications, and viral infections.^2^, ^3^, ^4^, ^5^, ^6^ Given the blaschkoid distribution, it’s possible that an acquired stimulus such as a virus or vaccination may induce loss of immunotolerance toward mosaic keratinocyte clones that undergo attack from, and clearance by, cytotoxic T cells. The above case suggests that multifactorial immune stimulation, in the setting of immunotherapy and COVID-19 infection, may have contributed to the etiology of this poorly understood dermatosis.

Importantly, previous reports have shown a link between COVID-19 and LS. For example, a 42-year-old woman developed LS 3 days after her second COVID vaccine.^2^ In another case, a previously vaccinated 24-year-old woman developed LS 2 days after developing COVID-19 symptoms.^5^ Our patient developed LS roughly 5 days after she tested positive for COVID-19 (and 9 days after initial symptoms). This timeline fits with the above literature; however, our patient also received treatment with ipilimumab and nivolumab just 2 days before developing LS.

LS has been linked to immunomodulatory medications in the past. For example, a 64-year-old man developed LS after his 24th week of treatment with interferon (IFN).^3^ LS has also been linked to tumor necrosis factor alpha (TNFα) inhibitors, etanercept and adalimumab.^4^ This may seem counterintuitive at first, however, TNFα inhibition has been shown to increase IFN levels, suggesting a common trigger in these medication-associated cases of LS.^7^ It has been proposed that anti-TNF treatment prolongs type-I IFN production by plasmacytoid dendritic cells through inhibition of their maturation.^7^ This theory is supported by another study which found that patients treated with TNF antagonists demonstrated overexpression of IFN-alpha regulated genes in peripheral blood leukocytes.^8^ Ultimately, IFN likely induces an endogenous inflammatory response where cells along the lines of Blaschko develop a heightened sensitivity to IFN, leading to cutaneous reactions like LS.^3^ Although lichenoid reaction patterns are common with ICI, to our knowledge, there is only one report describing LS as an immune-related adverse event , and no previous reports of LS induced by ICI. In this case, a 52-year-old female with cholangiocarcinoma developed LS like changes 2 weeks after her initial chimeric antigen receptor T-cell (CAR-T) infusion directed against EGFR.^9^ Interestingly, this first infusion was associated with the highest elevation in CAR transgene copy number. This peak occurred 9 days after her initial infusion and LS appeared 3 days later. Subsequent CAR-T infusions, including combined CAR-T ICI infusions, did not result in comparable peak CAR-T transgene copy number and there were no additional LS-like symptoms identified. It is possible that the massive cytokines directly produced by the large number of CAR-T cells led to a similar inflammatory response and, subsequently, loss of immune tolerance.

The above case describes 2 distinct immunostimulants that may have been triggers for the disease. Our patient developed LS 2 days after her third ICI infusion and 5 days after testing positive for COVID-19. The initial COVID-19 infection may have led to an initial loss of immune tolerance. In parallel, immunotherapy likely up regulated the self-lymphocytic response, leading to unmasking of host keratinocytes. Given the nature of this case however, it remains unclear whether only one of these events acted as the sole trigger or if they both ultimately contributed. Interestingly, other dermatologic manifestations, such as granulomatous reactions, have occurred in the setting of concurrent viral infections and immunotherapy.^10^

In conclusion, we report a unique case of adult-onset LS as an immune-related adverse event associated with immune checkpoint inhibitor use and concomitant COVID-19 infection. An already uncommon disease, reports of LS following these events are even rarer within the literature. Ultimately, more research is needed to investigate the role immune stimulation may play in the pathogenesis of this uncommon dermatologic condition.

## Conflicts of interest

Dr Gumbleton reports patents licensed to Alterna Therapeutics and honoraria from MJH Lifesciences and OMNI Health Media; all outside the scope of this publication. Drs Kelly, Bowen, Deacon, and Ms Kenyon have no conflicts of interest to declare.
